# Sennoside A+B Is as Effective as Polyethylene Glycol in Preparation for Small Intestine Capsule Endoscopy

**DOI:** 10.5152/tjg.2024.23398

**Published:** 2024-05-01

**Authors:** Fatma Ebru Akın, Öykü Tayfur Yürekli, Mustafa Tahtacı, Osman Ersoy

**Affiliations:** Department of Gastroenterology, Yıldırım Beyazıt University Faculty of Medicine, Ankara, Türkiye

**Keywords:** Bowel cleaning, Sennoside A+B, small bowel, video capsule endoscopy

## Abstract

**Background/Aims:**

We aimed to compare the effectiveness of the polyethylene glycol (PEG) and sennoside A+B regimens after clear fluid diet and fasting in bowel preperation of capsule endoscopy.

**Materials and Methods:**

In this retrospective single-center study, patients who were consecutively examined with small bowel capsule endoscopy (SBCE) between May 2010 and March 2023 were evaluated. Patients who underwent PEG 4 L and sennoside A+B calcium 250 mL for small bowel preparation were assigned. The quality of the small bowel cleaning and the diagnostic yield in detecting of small bowel lesions were compared.

**Results:**

Two hundred forty-two patients who underwent SBCE for various indications (PEG 74.4%, sennoside A+B 25.6%) were included in the study. The mean proximal small bowel cleaning scores was 1.97 ± 0.77 for PEG and 1.98 ± 0.04 (*P* = .83) for sennoside A+B; the mid small bowel cleaning scores was 1.76 ± 0.84 for PEG and 1.59 ± 0.05 (*P* = .108) for sennoside A+B; the mean distal small bowel cleaning scores was 1.27 ± 0.08 for PEG and 1.3 ± 0.54 (*P* = .805) for sennoside A+B; and the total small bowel cleaning scores was 1.66 ± 0.06 and 1.62 ± 0.04 (*P* = .622) for PEG and sennoside A+B, respectively. There were no significant differences regarding small bowel cleaning scores both segmentally and totally. At the same time, the diagnostic value of SBCE was similar in both groups.

**Conclusion:**

The effectiveness of sennoside A+B in SBCE preparation is similar to that of PEG and can be used in intestinal cleansing.

**Main Points**

- Before performing small bowel capsule endoscopy (SBCE), bowel cleansing is recommended to increase the diagnostic value of SBCE.
- It is recommended to use polyethylene glycol (PEG) for bowel preparation before performing SBCE.
- Sennoside A+B calcium also seems effective for small intestine cleansing before SBCE.
- The diagnostic value of SBCE appears to be similar to small bowel cleansing performed with sennoside A+B or PEG.

## Introduction

The small bowel capsule endoscopy (SBCE) has been used for the evaluating the small bowel for over 20 years. It has proven to be an important diagnostic method for detecting tumors or inflammation. Small bowel capsule endoscopy has been recommended as the first-line diagnostic method in the evaluation of obscure gastrointestinal bleeding (OGIB).^1^ Effective bowel cleaning is of utmost importance for optimizing the diagnostic role of SBCE. Blurred intestinal fluid, air bubbles, and food particles may impair the visibility of the small intestine and decrease the diagnostic yield.^2^ Nevertheless, the optimal bowel cleaning regimen has not been defined yet in the literature. Generally, overnight fasting (at least 12 hours) and clear fluid ingestion only are recommended. Despite the latest guidelines from the European Society of Gastrointestinal Endoscopy (ESGE) recommending the ingestion of a purgative [2 L of polyethylene glycol (PEG)] before SBCE for better visualization, the best pharmacotherapy to achieve better visualization and a higher diagnostic yield has not been universally agreed upon.^1,3^

Polyethylene glycol is a long-chain polymer of ethylene oxide, which acts as an osmotic laxative. Senna is a natural derivative of the plant senna. Sennosides A and B stimulate prostaglandin E2 production and secretion of chloride ions, causing changes in intestinal peristalsis and intraluminal water content.^4,5^ The American Gastroenterological Association recommends sennosides A and B for the patients with chronic idiopathic constipation. Compared with other bowel preparation regimens, the senna regimen may be effective and safe in bowel cleaning before colonoscopy, with superior compliance and tolerance.^5^ Yet, there are only a few studies evaluating the effectiveness of sennoside A+B calcium as a preparation regimen for SBCE. In this study, we compared the effectiveness of PEG and sennoside A+B calcium regimens for small bowel cleaning in the patients who underwent SBCE for various indications.

## Materials and Methods

Our study is a retrospective single-center study. Patients who previously underwent SBCE for various indications and received 4 L PEG (Golytely^®^) or 250 mL sennoside A+B calcium (X-M diet^®^) were included in this study. We used sennoside A+B as PEG was not available for some time in Türkiye, and sennoside A+B (X-M®) was covered by the reimbursement company during time period. All patients were recommended to ingest a clear fluid diet 1 day before the examination and fast from 18:00 until the examination time. Patients aged 18 and over who took 4 L of PEG or sennoside A+B calcium were included in this study. Patients under the age of 18, patients who used other drugs for bowel preparation, patients with a history of small bowel surgery, and patients in whom the capsule do not reach the cecum, were not included. Small bowel capsule endoscopy examinations were carried out using the PillCam(r) SB2 or SB3 capsule endoscopy system (Given Imaging, Yokneam, Israel). The primary outcome was the image quality of the small bowel images. The secondary outcome was the gastric and small bowel transit time. Another outcome measure was the rate of pathology detection. Since an improvement in bowel cleaning may increase the lesion detection, the rate of pathology detection was added as a tertiary criterion. The findings were considered diagnostic if the observed finding could explain the signs/symptoms of the patient, guide further management, or were later confirmed by other modalities. Patients who either used 4 L of PEG or sennoside A+B calcium were randomly assigned numbers, which were written on paper and placed in a bag. Later, papers were randomly selected from the bag, and the corresponding videos were evaluated by an expert gastroenterologist (F.E.A.), with experience interpreting over 350 SBCE cases. The expert noted gastric and small intestinal transit time and time to reach the cecum. The degree of bowel cleaning was classified according to Park’s classification.

### Scoring System (Park’s Classification)

**Figure 1.**
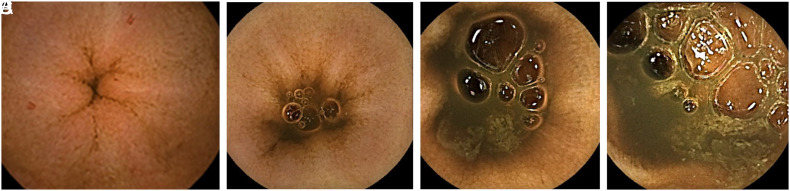
Images of Scores According to the Proportion of the Visualized Mucosa (A-D). A: Score 3; B: Score 2; C: Score 1; D: Score 0.

The first parameter was the ratio of visualized mucosa. This was scored based on a 4-step scale. Three points for greater than 75%, 2 points for between 50% and 75%, 1 point for between 25% and 50%, and 0 point for less than 25% (Figure 1).

**Figure 2.**
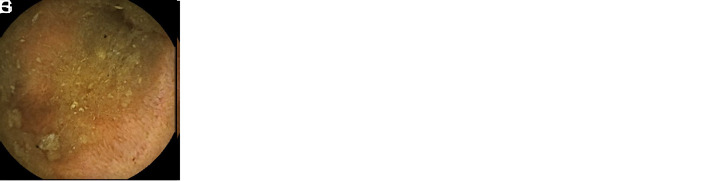
Images of Scores According to the Degree of Obscuration (E-H). E: Score 3; F: Score 2; G: Score 1; H: Score 0.

The second parameter was the degree of blurring with bubbles, residues, or bile. It was also evaluated on a 4-step scale based on 3 degrees: 3 points for less than 5% and no blurring, 2 points for mild blurring (from 5% to 25%), 1 point for moderate blurring (from 25% to 50%), and 0 point for severe blurring (greater than 50%) (Figure 2).

The time interval between the appearance of the first small intestinal image and the appearance of the cecum was defined as small bowel transit time. Then, the images between the first small intestinal image and the cecum image were selected serially at 5-minute intervals (1 square/5 minutes) by the RAPID system in manual mode. A different number of images were analyzed for each patient due to the differences in small intestinal transit time.

Mean points for each parameter were calculated by adding points of the selected images and then by dividing this number by the number of evaluated squares. Then the final for each parameter was calculated by the general mean value of these means.^6^ Obtained scores were separately calculated as proximal, mid, distal, and total by dividing them into 3, according to the transit time of the small bowel.

Since the study was retrospective and only the image records were analyzed, it was not necessary to obtain patient consent. Ethical approval was obtained from the Ankara City Hospital Ethics Committee No. 2 (July 20, 2022; E2-22-2156).

### Statistical Analysis

The collected sample and effect size were used to create a post hoc analysis of “observed power” following the analyses. When bowel cleaning scores were compared, it was discovered that the analysis had more power beyond the 0.80 cutoff point. Utilizing a 0.05 alpha level, 0.05 effect size, and sample sizes of preparation regimens sennoside and polyethylene glycol groups of 62 and 180, we discovered through post hoc G*Power analysis that the statistical power was 0.96.

Preliminary analyses were completed for descriptive, categorical, and continuous variables where applicable. The distribution properties of the data were evaluated with the Shapiro–Wilk test. Continuous variables were analyzed according to their distribution property using the Mann–Whitney *U* test or the independent *t* test. Statistical significance was set at *P* < .05 for all analyses. Statistical analysis was performed using SPSS version 17.0 (SPSS, Chicago, Ill, USA).

## Results

Three hundred thirty patients underwent video capsule endoscopies at Ankara Atatürk Hospital between May 2010 and January 2019, and at Ankara Bilkent City Hopsital between February 2019 and March 2023, for various indications in the study. Four patients under the age of 18, 33 patients who used other drugs for the bowel preparation, 4 patients who had small bowel surgery, and 39 patients in whom SBCE could not reach the cecum were excluded from the study. Additionally, 8 patients were also excluded due to unavailability of video capsule images. Finally, 242 patients were evaluated in the study. The mean age of the patients included in the study was 56.19 ± 1.05 with a female ratio of 39.3% (95 patients). Small bowel capsule endoscopy indications were obscure gastrointestinal bleeding, familial adenomatous polyposis coli, Behcet’s disease, Crohn’s disease, celiac disease, abdominal pain, and other reasons with the patient number and percentages, respectively: 197 (81.4%), 12 (5%), 2 (0.8%), 9 (3.7%), 3 (1.2%), 8 (3.3%), and 11 (4.5%). Sixty-two (25.6%) of the patients were given sennoside A+B calcium and 180 (74.4%) of them were given PEG as the preparation regimen (Table 1). The total number of evaluated images was 12 445, with a minimum image number per single video of 10, a maximum image number was 106, and a median image number was 49. Gastric transit, small bowel transit, and cecum transit times were 29.3 ± 2.35; 261.21 ± 5.53, and 291.97 ± 5.73 minutes, respectively. Mean proximal small bowel cleaning scores were 1.97 ± 0.77 and 1.98 ± 0.04 (*P* = .83) for PEG and sennoside A+B, respectively. Mean mid small bowel cleaning scores were 1.76 ± 0.84 and 1.59 ± 0.05 (*P* = .108) for PEG and sennoside A+B, respectively. Mean distal small bowel cleaning scores were 1.27 ± 0.08 and 1.3 ± 0.54 (*P* = .805) for PEG and sennoside A+B, respectively. Finally, the total small bowel cleaning scores were 1.66 ± 0.06 and 1.62 ± 0.04 (*P* = .622). There were no significant differences regarding small bowel cleaning scores both segmentally and totally (Table 2). Capsule endoscopic diagnoses in numbers and percentages were normal 88 (36.4), erosion 26 (10.7), ulcer 38 (15.7), vascular dysplasia 31 (12.3), polyp 9 (3.7), fresh blood 21 (8.7), tumor 10 (4.1), other 13 (5.4). The number and percentage of patients with predefined pathologies were 115 (63.9) (*P* = .889) and 39 (62.9), and there were no significant differences (Table 3).

## Discussion

This study reevaluating previously performed SBCE showed that sennoside A+B calcium and PEG performed similarly in terms of both bowel cleaning and diagnostic performance.

There is significant heterogeneity in the literature regarding the preparation regimens used prior to SBCE. Thus, the optimal regimen for bowel preparation has not been clearly defined yet.

There are numerous previous studies evaluating the role of bowel preparation for SBCE.^7^ Clinical practice varies from a clear liquid diet to the addition of purgatives like PEG to the clear liquid diet, but most of the literature supports the use of bowel preparation through PEG prior to the SBCE. In studies with PEG, various doses (ranging from 500 mL to 4 L) were adapted for use in capsule endoscopy, with contradictory results. Viazis et al randomized 80 patients to only a 2 L PEG and clear liquid diet group and found that the PEG group performed significantly better in terms of both adequate preparation and diagnostic efficacy.^8^ A meta-analysis of 982 patients from 9 randomized controlled trials concluded that SBCE after 2 L of PEG preparation increased the rate of small bowel imaging and hence diagnostic efficacy. Compared to 4 L of PEG, 2 L PEG resulted in similar imaging quality yet better tolerability.^9^ We compared 4 L PEG with sennosides A+B in our study. The reason for using 4 L PEG was the previous studies supporting 4 L PEG administration. In the study of 61 patients by Dai et al,^10^ 33 patients were given 4 L PEG while 28 patients were not given pre-procedure bowel preparation. They showed that bowel preparation accelerated the small bowel transit time with a higher rate of completion of the procedure.^10^ In our more recent video capsule endoscopies, we used sennosides A+B since PEG was not available in Türkiye, and sennosides A+B (X-M^®^) was covered by the reimbursement company. After analyzing the relevant 2 separate groups, we observed that sennosides A+B resulted in similar cleaning rates.

When we conducted a search for senna in the literature, we found that most reports evaluated its efficiency as a cleaning regimen prior to colonoscopy. Senna seems efficacious and safe, with excellent adherence and tolerance prior to colonoscopy.^5^ Our experience with senna as part of the cleaning regimen prior to colonoscopy also suggests excellent tolerability. There are limited studies that have used senna for small bowel cleaning prior to the SBCE.^11,12^ A randomized controlled trial by Chen et al^11^ included 180 patients and compared senna with mannitol. They reported that senna provided optimal small bowel cleaning and increased the diagnostic value of the study. These patients were asked to fast for 3 days, and the main side effects were fatigue and a feeling of hunger. A prospective single-blind randomized controlled study by Postgate et al^12^ included 150 patients. Compared to fluid restriction and 12 hours of fasting before the procedure, there was no additional benefit regarding procedure completion rates, and senna decreased patient adherence. In this study, patients were given questionnaires, and senna was found to be less comfortable when applied than the clear diet. However, patients were also willing to use the medication again because it would improve image quality.^12^ We detected that senna resulted in optimal small bowel cleaning rates.

In our study, gastric transit time was significantly shorter in the group that underwent bowel preparation with PEG. In the study conducted by Firemen et al,^13^ the effects of sodium phosphate and PEG on gastric and small intestine transit times were compared. Gastric transit time was observed to be shorter in the PEG group compared to both the sodium phosphate group and the control group.^13^ Although PEG appeared to shorten the gastric transit time, we found that there was no difference in the time to reach the cecum, bowel cleansing score, and diagnostic value. As a result, we believe that the shortening of gastric transit time is not significant in terms of positive clinical results.

Although some investigators have reported that bowel preparations such as PEG increased passage beyond the ileocecal valve (ICV),^10,13^ we observed no significant differences in rates of the passage beyond the ICV according to the preparation method. Our study also found no significant differences regarding small bowel transit times.

Optimal bowel cleaning before capsule endoscopy may improve the imaging of the small bowel, thus increasing the diagnostic value. Viazis et al indicated that bowel preparation with 2 L PEG increased the diagnostic value of SBCE.^8^ A meta-analysis stated that bowel cleaning improved the imaging quality and diagnostic value compared to the clear liquid diet.^7^ However, we found no study comparing PEG and senna regarding the diagnostic performance before small bowel capsule endoscopy. Our study indicated that both preparation regimens resulted in similar diagnosis rates, and bowel preparation with senna was not inferior to the PEG.

The first and probably most important limitation of our study is that it is a retrospective. The small size of the study groups may have prevented the detection of small differences between the preparation regimens. There are 2 experts with enough experience in our center, only one expert agreed to participate in the study. We did not exclude patients with diabetes and hyperthyroidism, which may affect the motility of the small bowel. Similarly, patients with constipation and hospitalized patients were not excluded, potentially leading to inadequate bowel cleaning due to the low bowel motility. Additionally, other drugs taken by the patients, which may alter the effect of the purgatives, were not analyzed. Besides, since it is a retrospective study, we could not define how purgatives affect the tolerability and adherence of the patients. Another limitation is that the relevant score may be negatively affected due to the continuing bleeding after the use of purgatives in patients with active small bowel bleeding. Finally, the lack of a universally approved SBCE quality scale and the inevitable long video recordings cause difficulties in evaluating the efficiency of interventions. This is a shared limitation of all the studies in this field.

The major strength of our study was its single-center study. Only one physician gave the recipes for bowel preparation before SBCE. This standardized the bowel preparation regimens.

As a result, we observed that sennoside A+B prior to SBCE is equally effective compared to PEG in terms of transit time, bowel cleaning, and diagnostic value. We believe sennoside A+B can be used as a bowel preparation regimen before SBCE, but further prospective and randomized studies are needed to evaluate the quality of cleaning and diagnostic yield.

## Figures and Tables

**Table 1.** Demographic and Clinical Data of the Patients

	Sennoside A+B	PEG	*P*	Total = 242, n (%)
Gender (N, %)				
Female	23	72	.4	95 (39.3)
Male	39	108	.4	147 (60.7)
Age	53.41 ± 2.25	57.14 ± 1.1	.13	
Indication (n, %)				
OGIB	52 (83.9)	145 (80.6)	.36	197 (81.5)
FAP	2 (3.2)	10 (5.6)	.37	12 (5)
Behcet’s disease	1 (1.6)	1 (0.6)	.45	2 (0.8)
Crohn disease	2 (3.2)	7 (3.9)	.58	9 (3.7)
Celiac disease	1 (1.6)	2 (1.1)	.59	3 (1.2)
Abdominal pain	1 (1.6)	7 (3.9)	.35	8 (3.3)
Other reasons	3 (4.8)	8 (4.4)	.57	11 (4.5)
Transit time (min)				
Gastric	39.11 ± 5.25	25.93 ± 2.56	.03	29.30 ± 2.35
Small bowel	251.11 ± 9.83	264.68 ± 6.62	.29	261.21 ± 5.53
Time to reach cecum	292.90 ± 10.21	291.65 ± 6.88	.93	291.97 ± 5.73

FAP; familial adenomatous polyposis; OGIB, obscure gastrointestinal bleeding; polyethylene glycol.

**Table 2.** Cleaning Scores for Small Bowel Segments

Small Bowel Segment	PEG Group	Sennoside A+B Group	*P*
Proximal	1.97 ± 0.77	1.98 ± 0.04	.83
Medium	1.76 ± 0.84	1.59 ± 0.05	.108
Distal	1.27 ± 0.08	1.3 ± 0.54	.805
TOTAL	1.66 ± 0.06	1.62 ± 0.04	.622

**Table 3.** Lesion Detected by SBCE and Percentages

Lesion	PEG Group (n, %)	Sennoside A+B Group (n, %)	Total (n, %)
Normal	65 (36.1)	23 (37.1)	88 (36.4)
Erosion	21 (11.7)	5 (8.1)	26 (10.7)
Ulcer	26 (14.4)	12 (19.4)	38 (15.7)
Angiodysplasia	26 (14.4)	5 (8.1)	31 (12.3)
Polyp	7 (3.9)	2 (3.2)	9 (3.7)
Fresh blood	17 (9.4)	4 (6.5)	21 (8.7)
Tumor	6 (3.3)	4 (6.5)	10 (4.1)
Polyp and angiodysplasia	2 (1.2)	0 (0)	2 (0.8)
Other	8 (4.4)	5 (8.1)	13 (5.4)
Erosion and angiodysplasia	2 (1.2)	1 (1.5)	3 (1.2)
Fresh blood and erosion	0	1 (1.5)	1 (0.4)

SBCE, small bowel capsule endoscopy.
